# *catena*-Poly[[chlorido(1,10-phenanthro­line)copper(II)]-μ-{2-[(1*S*,3*S*)-3-acetyl-2,2-dimethyl­cyclo­but­yl]acetato}]

**DOI:** 10.1107/S1600536811048586

**Published:** 2011-11-19

**Authors:** Shi-ying Ma, Yan-fei Li

**Affiliations:** a Department of Chemistry and Environmental Sciences, Taishan University, 271021 Taian, Shandong, People’s Republic of China; b Department of Materials and Chemistry Engineering, Taishan University, 271021 Taian, Shandong, People’s Republic of China

## Abstract

The title compound, [Cu(C_10_H_15_O_3_)Cl(C_12_H_8_N_2_)]_*n*_, is a one-dimensional coordination polymer. The Cu^II^ atom is coordin­ated by a chloride ion, two N atoms from the 1,10-phenanthroline ligand, and a monodentate carboxyl­ate O atom from the pinononate anion, forming a CuN_2_ClO approximate square plane. A symmetry-generated pinononate O atom completes a square-based pyramidal geometry for the copper ion. The bridging 2-(3-acetyl-2,2-dimethyl­cyclo­but­yl)acetate anion leads to chains in the crystal propagating in [001]. Adjacent 1,10-phenanthroline rings form a dihedral angle of 39.4 (2)°.

## Related literature

For related structures, see: Che *et al.* (2006 ▶); Lalancette *et al.* (1999 ▶); Vanderhoff *et al.* (1986 ▶). 
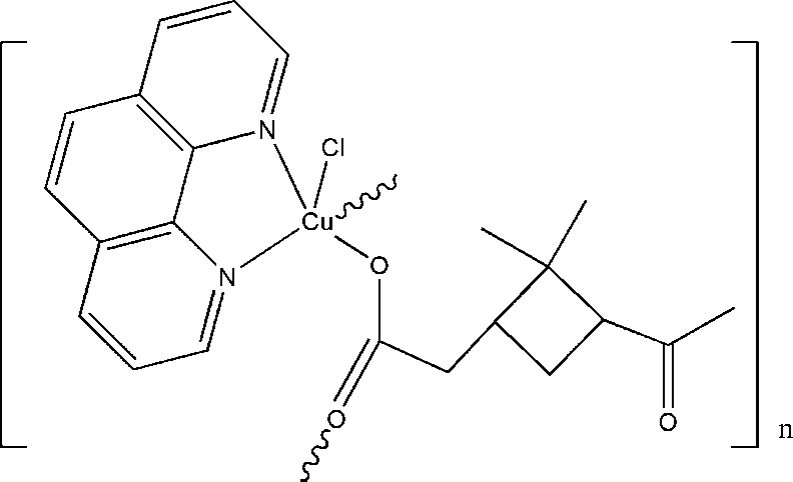


## Experimental

### 

#### Crystal data

[Cu(C_10_H_15_O_3_)Cl(C_12_H_8_N_2_)]
*M* *_r_* = 462.41
Monoclinic, 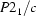

*a* = 14.6143 (11) Å
*b* = 14.4920 (12) Å
*c* = 9.8419 (8) Å
β = 98.208 (1)°
*V* = 2063.1 (3) Å^3^
*Z* = 4
Mo *K*α radiation
μ = 1.21 mm^−1^
*T* = 273 K
0.20 × 0.18 × 0.16 mm

#### Data collection

Siemens SMART CCD diffractometer
Absorption correction: multi-scan (*SADABS*; Siemens, 1996 ▶) *T*_min_ = 0.793, *T*_max_ = 0.829
10533 measured reflections
3647 independent reflections
2924 reflections with *I* > 2σ(*I*)
*R*_int_ = 0.028

#### Refinement

*R*[*F* ^2^ > 2σ(*F* ^2^)] = 0.036
*wR*(*F* ^2^) = 0.097
*S* = 1.03
3647 reflections
265 parameters
H-atom parameters constrained
Δρ_max_ = 0.67 e Å^−3^
Δρ_min_ = −0.30 e Å^−3^

### 

Data collection: *SMART* (Siemens, 1996 ▶); cell refinement: *SAINT* (Siemens, 1996 ▶); data reduction: *SAINT*; program(s) used to solve structure: *SHELXS97* (Sheldrick, 2008 ▶); program(s) used to refine structure: *SHELXL97* (Sheldrick, 2008 ▶); molecular graphics: *SHELXTL* (Sheldrick, 2008 ▶); software used to prepare material for publication: *SHELXTL*.

## Supplementary Material

Crystal structure: contains datablock(s) global, I. DOI: 10.1107/S1600536811048586/hb6482sup1.cif

Structure factors: contains datablock(s) I. DOI: 10.1107/S1600536811048586/hb6482Isup2.hkl

Additional supplementary materials:  crystallographic information; 3D view; checkCIF report

## Figures and Tables

**Table 1:** Selected bond lengths (Å)

Cu1—O1	1.937 (2)
Cu1—N2	2.030 (2)
Cu1—N1	2.050 (2)
Cu1—Cl1	2.2610 (8)
Cu1—O2^i^	2.305 (2)

Symmetry code: (i) 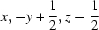.

**Fig. 1.**
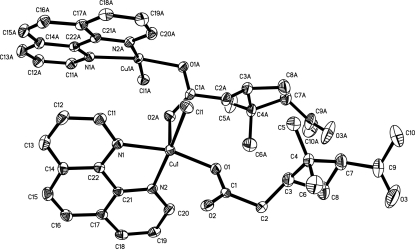
The molecular structure of (I), with displacement ellipsoids drawn at the 30% probability level.

## supplementary crystallographic information

### Comment

In the structural investigation of pinonate complexes, it has been found that the pinonic acid functions as a monodenate ligand (Lalancette *et al.*, 1999). We sythesized the pinonic acid that obtained from α-pinene oxidated by potassium permangate. In the present paper, we describe the crystal stucture of the title compound.The polymer molecule contains CuN_2_O_2_Cl square-based pyramids (Fig.1). The Cu(II) atom exists in a distorted square pyramidal enviroment,defined by two carboxyl O atoms from two monodentate pinonate ligand,two N atoms from 1,10-phenanthroline ligand and one Cl^-^ anion.

### Experimental

Pinonic acid is synthesized from α-pinene oxidated by potassium permangate.The pinonic acid (0.5 mmol)and NaOH (0.5 mmol) were dissolved in methanol (5 ml). A methanolic solution (5 ml) of CuCl_2_.H_2_O (0.25 mmol) and 1,10-phenanthroline (0.25 mmol) was slowly droped to the solution of the pinonic acid with stirring.The result solution was flitered and allowed to stand in air at room temperature for seven days,yielding blue blocks of (I).

### Refinement

All H atoms were initially located in a difference Fourier map and were placed in geometrically idealized positions, with C—H = 0.93 - 0.97 Å and *U*_iso_(H) = 1.2*U*_eq_(C).

### Crystal data

**Table:**

[Cu(C_10_H_15_O_3_)Cl(C_12_H_8_N_2_)]	*F*(000) = 956
*M**_r_* = 462.41	*D*_x_ = 1.489 Mg m^−^^3^
Monoclinic, *P*2_1_/*c*	Mo *K*α radiation, λ = 0.71073 Å
Hall symbol: -P 2ybc	Cell parameters from 3334 reflections
*a* = 14.6143 (11) Å	θ = 2.5–25.1°
*b* = 14.4920 (12) Å	µ = 1.21 mm^−^^1^
*c* = 9.8419 (8) Å	*T* = 273 K
β = 98.208 (1)°	Block, blue
*V* = 2063.1 (3) Å^3^	0.20 × 0.18 × 0.16 mm
*Z* = 4	

### Data collection

**Table:**

Siemens SMART CCD diffractometer	3647 independent reflections
Radiation source: fine-focus sealed tube	2924 reflections with *I* > 2σ(*I*)
graphite	*R*_int_ = 0.028
phi and ω scans	θ_max_ = 25.1°, θ_min_ = 1.4°
Absorption correction: multi-scan (*SADABS*; Siemens, 1996)	*h* = −15→17
*T*_min_ = 0.793, *T*_max_ = 0.829	*k* = −17→14
10533 measured reflections	*l* = −11→11

### Refinement

**Table:**

Refinement on *F*^2^	Primary atom site location: structure-invariant direct methods
Least-squares matrix: full	Secondary atom site location: difference Fourier map
*R*[*F*^2^ > 2σ(*F*^2^)] = 0.036	Hydrogen site location: inferred from neighbouring sites
*wR*(*F*^2^) = 0.097	H-atom parameters constrained
*S* = 1.03	*w* = 1/[σ^2^(*F*_o_^2^) + (0.0467*P*)^2^ + 1.5378*P*] where *P* = (*F*_o_^2^ + 2*F*_c_^2^)/3
3647 reflections	(Δ/σ)_max_ = 0.001
265 parameters	Δρ_max_ = 0.67 e Å^−^^3^
0 restraints	Δρ_min_ = −0.30 e Å^−^^3^

### Special details

**Table:**

Geometry. All e.s.d.'s (except the e.s.d. in the dihedral angle between two l.s. planes)are estimated using the full covariance matrix. The cell e.s.d.'s are takeninto account individually in the estimation of e.s.d.'s in distances, anglesand torsion angles; correlations between e.s.d.'s in cell parameters are onlyused when they are defined by crystal symmetry. An approximate (isotropic)treatment of cell e.s.d.'s is used for estimating e.s.d.'s involving l.s. planes.
Refinement. Refinement of *F*^2^ against ALL reflections. The weighted *R*-factor *wR* and goodness of fit *S* are based on *F*^2^, conventional *R*-factors *R* are based on *F*, with *F* set to zero for negative *F*^2^. The threshold expression of *F*^2^ > σ(*F*^2^) is used only for calculating *R*-factors(gt) *etc*. and is not relevant to the choice of reflections for refinement. *R*-factors based on *F*^2^ are statistically about twice as large as those based on *F*, and *R*- factors based on ALL data will be even larger.

### Fractional atomic coordinates and isotropic or equivalent isotropic displacement parameters (Å^2^)

**Table:**

	*x*	*y*	*z*	*U*_iso_*/*U*_eq_	
Cu1	0.38042 (2)	0.23582 (2)	0.56377 (4)	0.02863 (13)	
Cl1	0.34460 (5)	0.08697 (5)	0.51189 (8)	0.0398 (2)	
O1	0.26729 (14)	0.25754 (14)	0.6401 (2)	0.0347 (5)	
O2	0.33639 (14)	0.21019 (14)	0.8438 (2)	0.0355 (5)	
O3	−0.1289 (3)	0.1493 (3)	0.8971 (5)	0.1157 (15)	
N1	0.51618 (16)	0.22377 (16)	0.5353 (2)	0.0289 (5)	
N2	0.42648 (16)	0.36312 (16)	0.6269 (2)	0.0295 (5)	
C1	0.26816 (19)	0.24062 (19)	0.7671 (3)	0.0279 (6)	
C2	0.1786 (2)	0.2630 (2)	0.8208 (3)	0.0397 (8)	
H2A	0.1808	0.2364	0.9116	0.048*	
H2B	0.1739	0.3294	0.8298	0.048*	
C3	0.0939 (2)	0.2285 (2)	0.7320 (4)	0.0461 (8)	
H3	0.0985	0.2437	0.6362	0.055*	
C4	0.0640 (2)	0.1255 (3)	0.7399 (3)	0.0435 (8)	
C5	0.0936 (3)	0.0622 (3)	0.6319 (5)	0.0767 (14)	
H5A	0.1598	0.0615	0.6401	0.115*	
H5B	0.0683	0.0842	0.5423	0.115*	
H5C	0.0714	0.0010	0.6447	0.115*	
C6	0.0917 (3)	0.0859 (3)	0.8825 (4)	0.0663 (11)	
H6A	0.0653	0.0256	0.8872	0.099*	
H6B	0.0695	0.1255	0.9489	0.099*	
H6C	0.1578	0.0817	0.9016	0.099*	
C7	−0.0376 (2)	0.1627 (3)	0.7195 (4)	0.0574 (10)	
H7	−0.0622	0.1633	0.6214	0.069*	
C8	−0.0020 (3)	0.2584 (3)	0.7638 (5)	0.0651 (11)	
H8A	−0.0037	0.2718	0.8600	0.078*	
H8B	−0.0299	0.3079	0.7057	0.078*	
C9	−0.1054 (3)	0.1142 (3)	0.7988 (5)	0.0697 (12)	
C10	−0.1413 (3)	0.0230 (4)	0.7437 (6)	0.0972 (18)	
H10A	−0.1668	−0.0102	0.8139	0.146*	
H10B	−0.0916	−0.0121	0.7153	0.146*	
H10C	−0.1884	0.0326	0.6665	0.146*	
C11	0.5589 (2)	0.1529 (2)	0.4869 (3)	0.0375 (7)	
H11	0.5255	0.0995	0.4619	0.045*	
C12	0.6521 (2)	0.1559 (2)	0.4722 (4)	0.0451 (8)	
H12	0.6801	0.1048	0.4383	0.054*	
C13	0.7023 (2)	0.2334 (2)	0.5073 (4)	0.0458 (8)	
H13	0.7645	0.2358	0.4968	0.055*	
C14	0.6600 (2)	0.3100 (2)	0.5596 (3)	0.0357 (7)	
C15	0.7052 (2)	0.3956 (2)	0.5989 (3)	0.0435 (8)	
H15	0.7678	0.4019	0.5929	0.052*	
C16	0.6593 (2)	0.4668 (2)	0.6442 (3)	0.0411 (8)	
H16	0.6907	0.5214	0.6695	0.049*	
C17	0.5629 (2)	0.4600 (2)	0.6541 (3)	0.0330 (7)	
C18	0.5098 (2)	0.5331 (2)	0.6977 (3)	0.0370 (7)	
H18	0.5367	0.5901	0.7208	0.044*	
C19	0.4190 (2)	0.5183 (2)	0.7050 (3)	0.0406 (8)	
H19	0.3831	0.5656	0.7336	0.049*	
C20	0.3791 (2)	0.4324 (2)	0.6697 (3)	0.0367 (7)	
H20	0.3169	0.4236	0.6765	0.044*	
C21	0.51761 (19)	0.37713 (19)	0.6199 (3)	0.0275 (6)	
C22	0.56575 (19)	0.3011 (2)	0.5705 (3)	0.0295 (6)	

### Atomic displacement parameters (Å^2^)

**Table:**

	*U*^11^	*U*^22^	*U*^33^	*U*^12^	*U*^13^	*U*^23^
Cu1	0.0292 (2)	0.0275 (2)	0.0297 (2)	−0.00360 (15)	0.00622 (14)	−0.00034 (15)
Cl1	0.0446 (5)	0.0285 (4)	0.0455 (5)	−0.0070 (3)	0.0040 (4)	−0.0003 (3)
O1	0.0312 (11)	0.0448 (13)	0.0287 (11)	−0.0034 (9)	0.0060 (9)	0.0014 (9)
O2	0.0377 (12)	0.0373 (12)	0.0309 (12)	0.0077 (9)	0.0032 (9)	−0.0031 (9)
O3	0.109 (3)	0.110 (3)	0.152 (4)	−0.012 (2)	0.098 (3)	−0.022 (3)
N1	0.0325 (13)	0.0249 (13)	0.0299 (13)	−0.0009 (10)	0.0064 (10)	−0.0007 (10)
N2	0.0319 (13)	0.0291 (13)	0.0285 (13)	−0.0023 (11)	0.0074 (10)	−0.0016 (10)
C1	0.0291 (15)	0.0235 (15)	0.0319 (16)	−0.0071 (12)	0.0071 (13)	−0.0064 (12)
C2	0.0345 (17)	0.051 (2)	0.0347 (18)	−0.0008 (15)	0.0101 (13)	−0.0085 (15)
C3	0.0356 (18)	0.060 (2)	0.044 (2)	−0.0002 (16)	0.0080 (15)	0.0042 (17)
C4	0.0289 (17)	0.058 (2)	0.045 (2)	−0.0049 (15)	0.0095 (14)	−0.0051 (17)
C5	0.058 (3)	0.095 (3)	0.082 (3)	−0.023 (2)	0.026 (2)	−0.043 (3)
C6	0.067 (3)	0.061 (3)	0.071 (3)	0.004 (2)	0.012 (2)	0.010 (2)
C7	0.0360 (19)	0.075 (3)	0.061 (2)	−0.0026 (19)	0.0078 (17)	0.004 (2)
C8	0.042 (2)	0.066 (3)	0.088 (3)	0.0026 (19)	0.011 (2)	0.007 (2)
C9	0.039 (2)	0.090 (3)	0.085 (3)	−0.013 (2)	0.026 (2)	−0.003 (3)
C10	0.065 (3)	0.107 (4)	0.122 (5)	−0.044 (3)	0.023 (3)	−0.006 (3)
C11	0.0436 (18)	0.0303 (17)	0.0400 (18)	−0.0009 (14)	0.0106 (14)	−0.0020 (14)
C12	0.0455 (19)	0.0368 (19)	0.057 (2)	0.0080 (16)	0.0200 (17)	−0.0022 (16)
C13	0.0365 (18)	0.045 (2)	0.059 (2)	0.0019 (16)	0.0186 (16)	0.0034 (17)
C14	0.0311 (16)	0.0359 (17)	0.0411 (18)	−0.0034 (14)	0.0089 (13)	0.0024 (14)
C15	0.0353 (18)	0.045 (2)	0.052 (2)	−0.0086 (15)	0.0119 (15)	0.0008 (16)
C16	0.0419 (18)	0.0379 (19)	0.0434 (19)	−0.0153 (15)	0.0062 (15)	−0.0018 (15)
C17	0.0392 (17)	0.0322 (17)	0.0272 (16)	−0.0054 (13)	0.0040 (13)	0.0002 (13)
C18	0.050 (2)	0.0267 (16)	0.0332 (17)	−0.0053 (14)	0.0038 (14)	−0.0042 (13)
C19	0.052 (2)	0.0323 (18)	0.0387 (19)	0.0045 (15)	0.0121 (15)	−0.0070 (14)
C20	0.0362 (17)	0.0362 (18)	0.0396 (18)	0.0023 (14)	0.0118 (14)	−0.0026 (14)
C21	0.0322 (15)	0.0284 (15)	0.0221 (15)	−0.0039 (12)	0.0044 (11)	−0.0008 (12)
C22	0.0325 (15)	0.0269 (15)	0.0289 (15)	−0.0020 (13)	0.0041 (12)	0.0011 (12)

### Geometric parameters (Å, °)

**Table:**

Cu1—O1	1.937 (2)	C7—C9	1.519 (5)
Cu1—N2	2.030 (2)	C7—C8	1.523 (6)
Cu1—N1	2.050 (2)	C7—H7	0.9800
Cu1—Cl1	2.2610 (8)	C8—H8A	0.9700
Cu1—O2^i^	2.305 (2)	C8—H8B	0.9700
O1—C1	1.272 (4)	C9—C10	1.496 (7)
O2—C1	1.242 (3)	C10—H10A	0.9600
O2—Cu1^ii^	2.305 (2)	C10—H10B	0.9600
O3—C9	1.186 (5)	C10—H10C	0.9600
N1—C11	1.326 (4)	C11—C12	1.391 (4)
N1—C22	1.352 (4)	C11—H11	0.9300
N2—C20	1.322 (4)	C12—C13	1.360 (5)
N2—C21	1.359 (3)	C12—H12	0.9300
C1—C2	1.514 (4)	C13—C14	1.403 (4)
C2—C3	1.496 (5)	C13—H13	0.9300
C2—H2A	0.9700	C14—C22	1.403 (4)
C2—H2B	0.9700	C14—C15	1.431 (4)
C3—C8	1.541 (5)	C15—C16	1.342 (5)
C3—C4	1.559 (5)	C15—H15	0.9300
C3—H3	0.9800	C16—C17	1.430 (4)
C4—C5	1.513 (5)	C16—H16	0.9300
C4—C6	1.517 (5)	C17—C21	1.390 (4)
C4—C7	1.566 (5)	C17—C18	1.414 (4)
C5—H5A	0.9600	C18—C19	1.357 (4)
C5—H5B	0.9600	C18—H18	0.9300
C5—H5C	0.9600	C19—C20	1.398 (4)
C6—H6A	0.9600	C19—H19	0.9300
C6—H6B	0.9600	C20—H20	0.9300
C6—H6C	0.9600	C21—C22	1.430 (4)
			
O1—Cu1—N2	89.87 (9)	C9—C7—H7	109.7
O1—Cu1—N1	164.36 (9)	C8—C7—H7	109.7
N2—Cu1—N1	80.45 (9)	C4—C7—H7	109.7
O1—Cu1—Cl1	93.42 (6)	C7—C8—C3	88.2 (3)
N2—Cu1—Cl1	172.71 (7)	C7—C8—H8A	113.9
N1—Cu1—Cl1	94.89 (7)	C3—C8—H8A	113.9
O1—Cu1—O2^i^	99.78 (8)	C7—C8—H8B	113.9
N2—Cu1—O2^i^	90.84 (8)	C3—C8—H8B	113.9
N1—Cu1—O2^i^	92.67 (8)	H8A—C8—H8B	111.1
Cl1—Cu1—O2^i^	95.00 (6)	O3—C9—C10	123.2 (4)
C1—O1—Cu1	117.42 (18)	O3—C9—C7	120.5 (4)
C1—O2—Cu1^ii^	122.84 (18)	C10—C9—C7	116.3 (4)
C11—N1—C22	118.1 (2)	C9—C10—H10A	109.5
C11—N1—Cu1	129.0 (2)	C9—C10—H10B	109.5
C22—N1—Cu1	112.83 (18)	H10A—C10—H10B	109.5
C20—N2—C21	117.7 (3)	C9—C10—H10C	109.5
C20—N2—Cu1	128.4 (2)	H10A—C10—H10C	109.5
C21—N2—Cu1	113.88 (18)	H10B—C10—H10C	109.5
O2—C1—O1	124.1 (3)	N1—C11—C12	122.1 (3)
O2—C1—C2	121.5 (3)	N1—C11—H11	118.9
O1—C1—C2	114.4 (3)	C12—C11—H11	118.9
C3—C2—C1	114.2 (3)	C13—C12—C11	120.0 (3)
C3—C2—H2A	108.7	C13—C12—H12	120.0
C1—C2—H2A	108.7	C11—C12—H12	120.0
C3—C2—H2B	108.7	C12—C13—C14	119.9 (3)
C1—C2—H2B	108.7	C12—C13—H13	120.1
H2A—C2—H2B	107.6	C14—C13—H13	120.1
C2—C3—C8	119.2 (3)	C22—C14—C13	116.3 (3)
C2—C3—C4	120.3 (3)	C22—C14—C15	118.6 (3)
C8—C3—C4	89.5 (3)	C13—C14—C15	125.0 (3)
C2—C3—H3	108.8	C16—C15—C14	121.6 (3)
C8—C3—H3	108.8	C16—C15—H15	119.2
C4—C3—H3	108.8	C14—C15—H15	119.2
C5—C4—C6	110.8 (4)	C15—C16—C17	120.9 (3)
C5—C4—C3	115.8 (3)	C15—C16—H16	119.6
C6—C4—C3	111.6 (3)	C17—C16—H16	119.6
C5—C4—C7	118.6 (3)	C21—C17—C18	117.2 (3)
C6—C4—C7	111.8 (3)	C21—C17—C16	118.9 (3)
C3—C4—C7	86.1 (3)	C18—C17—C16	123.9 (3)
C4—C5—H5A	109.5	C19—C18—C17	118.8 (3)
C4—C5—H5B	109.5	C19—C18—H18	120.6
H5A—C5—H5B	109.5	C17—C18—H18	120.6
C4—C5—H5C	109.5	C18—C19—C20	120.3 (3)
H5A—C5—H5C	109.5	C18—C19—H19	119.9
H5B—C5—H5C	109.5	C20—C19—H19	119.9
C4—C6—H6A	109.5	N2—C20—C19	122.4 (3)
C4—C6—H6B	109.5	N2—C20—H20	118.8
H6A—C6—H6B	109.5	C19—C20—H20	118.8
C4—C6—H6C	109.5	N2—C21—C17	123.6 (3)
H6A—C6—H6C	109.5	N2—C21—C22	115.8 (2)
H6B—C6—H6C	109.5	C17—C21—C22	120.6 (3)
C9—C7—C8	119.7 (4)	N1—C22—C14	123.6 (3)
C9—C7—C4	116.8 (3)	N1—C22—C21	117.0 (2)
C8—C7—C4	89.8 (3)	C14—C22—C21	119.4 (3)
			
N2—Cu1—O1—C1	−84.8 (2)	C4—C3—C8—C7	−19.2 (3)
N1—Cu1—O1—C1	−33.3 (4)	C8—C7—C9—O3	−1.9 (7)
Cl1—Cu1—O1—C1	88.70 (19)	C4—C7—C9—O3	104.6 (5)
O2^i^—Cu1—O1—C1	−175.62 (19)	C8—C7—C9—C10	176.5 (4)
O1—Cu1—N1—C11	128.7 (3)	C4—C7—C9—C10	−76.9 (5)
N2—Cu1—N1—C11	−178.8 (3)	C22—N1—C11—C12	0.3 (4)
Cl1—Cu1—N1—C11	6.8 (3)	Cu1—N1—C11—C12	179.2 (2)
O2^i^—Cu1—N1—C11	−88.4 (3)	N1—C11—C12—C13	−0.4 (5)
O1—Cu1—N1—C22	−52.3 (4)	C11—C12—C13—C14	0.6 (5)
N2—Cu1—N1—C22	0.14 (19)	C12—C13—C14—C22	−0.8 (5)
Cl1—Cu1—N1—C22	−174.20 (18)	C12—C13—C14—C15	−179.4 (3)
O2^i^—Cu1—N1—C22	90.55 (19)	C22—C14—C15—C16	−0.6 (5)
O1—Cu1—N2—C20	−13.9 (3)	C13—C14—C15—C16	177.9 (3)
N1—Cu1—N2—C20	178.4 (3)	C14—C15—C16—C17	−0.4 (5)
Cl1—Cu1—N2—C20	−130.9 (5)	C15—C16—C17—C21	1.9 (5)
O2^i^—Cu1—N2—C20	85.9 (3)	C15—C16—C17—C18	−178.3 (3)
O1—Cu1—N2—C21	167.71 (19)	C21—C17—C18—C19	0.7 (4)
N1—Cu1—N2—C21	0.05 (19)	C16—C17—C18—C19	−179.1 (3)
Cl1—Cu1—N2—C21	50.7 (6)	C17—C18—C19—C20	−0.1 (5)
O2^i^—Cu1—N2—C21	−92.51 (19)	C21—N2—C20—C19	1.1 (4)
Cu1^ii^—O2—C1—O1	137.8 (2)	Cu1—N2—C20—C19	−177.3 (2)
Cu1^ii^—O2—C1—C2	−40.6 (3)	C18—C19—C20—N2	−0.8 (5)
Cu1—O1—C1—O2	−0.9 (4)	C20—N2—C21—C17	−0.5 (4)
Cu1—O1—C1—C2	177.59 (19)	Cu1—N2—C21—C17	178.1 (2)
O2—C1—C2—C3	−134.8 (3)	C20—N2—C21—C22	−178.8 (3)
O1—C1—C2—C3	46.7 (4)	Cu1—N2—C21—C22	−0.2 (3)
C1—C2—C3—C8	−171.3 (3)	C18—C17—C21—N2	−0.4 (4)
C1—C2—C3—C4	80.3 (4)	C16—C17—C21—N2	179.4 (3)
C2—C3—C4—C5	−97.2 (4)	C18—C17—C21—C22	177.9 (3)
C8—C3—C4—C5	138.7 (4)	C16—C17—C21—C22	−2.4 (4)
C2—C3—C4—C6	30.8 (4)	C11—N1—C22—C14	−0.6 (4)
C8—C3—C4—C6	−93.2 (3)	Cu1—N1—C22—C14	−179.6 (2)
C2—C3—C4—C7	142.8 (3)	C11—N1—C22—C21	178.8 (3)
C8—C3—C4—C7	18.7 (3)	Cu1—N1—C22—C21	−0.3 (3)
C5—C4—C7—C9	100.1 (5)	C13—C14—C22—N1	0.8 (5)
C6—C4—C7—C9	−30.8 (5)	C15—C14—C22—N1	179.5 (3)
C3—C4—C7—C9	−142.6 (4)	C13—C14—C22—C21	−178.5 (3)
C5—C4—C7—C8	−136.3 (4)	C15—C14—C22—C21	0.2 (4)
C6—C4—C7—C8	92.8 (4)	N2—C21—C22—N1	0.4 (4)
C3—C4—C7—C8	−19.0 (3)	C17—C21—C22—N1	−178.0 (3)
C9—C7—C8—C3	140.3 (4)	N2—C21—C22—C14	179.7 (3)
C4—C7—C8—C3	19.2 (3)	C17—C21—C22—C14	1.4 (4)
C2—C3—C8—C7	−144.2 (3)		

Symmetry codes: (i) *x*, −*y*+1/2, *z*−1/2; (ii) *x*, −*y*+1/2, *z*+1/2.
